# A Capsule-Type Electromagnetic Acoustic Transducer for Fast Screening of External Corrosion in Nonmagnetic Pipes

**DOI:** 10.3390/s18061733

**Published:** 2018-05-28

**Authors:** Yong Li, Rui Cai, Bei Yan, Ilham Mukriz Zainal Abidin, Haoqing Jing, Yi Wang

**Affiliations:** 1 State Key Laboratory for Strength and Vibration of Mechanical Structures, Shaanxi Engineering Research Center of NDT and Structural Integrity Evaluation, School of Aerospace Engineering, Xi’an Jiaotong University, Xi’an 710049, China; cr739206859@stu.xjtu.edu.cn (R.C.); yanbei@stu.xjtu.edu.cn (B.Y.); jinghaoqing@stu.xjtu.edu.cn (H.J.); wybit2008@stu.xjtu.edu.cn (Y.W.); 2 Leading Edge NDT Technology (LENDT) Group, Malaysian Nuclear Agency, Bangi, Kajang 43000, Malaysia; mukriz@nuclearmalaysia.gov.my

**Keywords:** ultrasound transducer, electromagnetic acoustic transducer, nonmagnetic pipe, external corrosion, hybrid modeling

## Abstract

For fuel transmission and structural strengthening, small-diameter pipes of nonmagnetic materials are extensively adopted in engineering fields including aerospace, energy, transportation, etc. However, the hostile and corrosive environment leaves them vulnerable to external corrosion which poses a severe threat to structural integrity of pipes. Therefore, it is imperative to nondestructively detect and evaluate the external corrosion in nonmagnetic pipes. In light of this, a capsule-type Electromagnetic Acoustic Transducer (EMAT) for in-situ nondestructive evaluation of nonmagnetic pipes and fast screening of external corrosion is proposed in this paper. A 3D hybrid model for efficient prediction of responses from the proposed transducer to external corrosion is established. Closed-form expressions of field quantities of electromagnetics and EMAT signals are formulated. Simulations based on the hybrid model indicate feasibility of the proposed transducer in detection and evaluation of external corrosion in nonmagnetic pipes. In parallel, experiments with the fabricated transducer have been carried out. Experimental results are supportive of the conclusion drawn from simulations. The investigation via simulations and experiments implies that the proposed capsule-type EMAT is capable of fast screening of external corrosion, which is beneficial to the in-situ nondestructive evaluation of small-diameter nonmagnetic pipes.

## 1. Introduction

Metallic pipes of such nonmagnetic materials as aluminum and copper (i.e., nonmagnetic pipes) are widely employed in engineering structures in the fields of aerospace, energy, chemical, etc. As one of the critical components for fuel transmission and structural strengthening, in-service nonmagnetic pipes particularly with small diameters are vulnerable to the external corrosion which occurs in the outer surface of the pipe due to hostile and corrosive environment. Since such corrosion severely undermines the structural integrity of the pipes, it is indispensable to nondestructively detect and evaluate the external corrosion whose progress will lead to pipe failure and catastrophic accidents. Because of the rigorous inspection condition, probes of such traditional Nondestructive Testing (NDT) techniques as Eddy Current Testing (ECT) [1,2] and Radiography Testing (RT) [3] can barely be deployed over the pipe outer surface. This makes the external corrosion unreachable and undetected. Therefore, in-situ NDT is preferred with probes/transducers placed in the nonmagnetic pipe. Although NDT techniques such as Pulsed Eddy Current (PEC) [4] are found to be promising in in-situ NDT and evaluation of subsurface corrosion, it is intricate for them to inspect the nonmagnetic pipe with the thick wall over 10 mm. In light of these, Ultrasonic Testing (UT) [5,6,7] is preferred for detection and evaluation of external corrosion in nonmagnetic pipes. However, the traditional piezoelectric-crystal-based UT technique suffers from the technical drawbacks in terms of surface preparation needed and couplants demanded throughout the UT inspection. This hinders the application of UT in real-time and efficient inspection of in-service nonmagnetic pipes.

Electromagnetic acoustic transduction is one of the advanced UT techniques whilst it has the advantages over the traditional piezoelectric-crystal-based UT technique. It realizes contactless inspection without couplants, and thus can be applicable for high-speed detection and evaluation of flaws in metallic structures in high-temperature and high-stress environments [8,9,10]. However, the conventional Electromagnetic Acoustic Transducer (EMAT) is subject to low transduction efficiency resulting from the lift-off interference as the probe can hardly fully fit the inner surface of the pipe under inspection. Even though the lift-off interference could be mitigated by using the permanent magnet with curved surface at the pole together with the flexible coil [11], the inspection of nonmagnetic pipes is still prone to low inspection efficiency. The reasoning lies in the fact that the incident ultrasonic wave is locally excited, and thus only a section of the pipe along its circumference can be inspected. Therefore, 2D scanning in both circumferential and axial directions is highly demanded [12].

**Figure 1:**
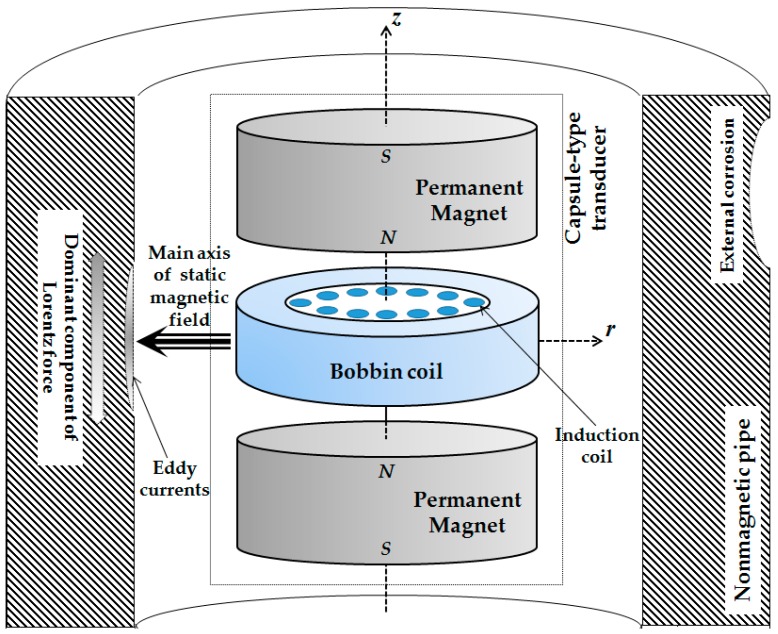
The proposed capsule-type Electromagnetic Acoustic Transducer (EMAT) for pipe inspection.

In light of this, a capsule-type EMAT for efficient screening of external corrosion in nonmagnetic pipes is proposed. The schematic illustration of the transducer is portrayed in Figure 1. It comprises a pair of cylindrical magnets, a bobbin coil and array of induction coils. The concentric magnet pair is in repulsive configuration with same poles facing to each other. It is adopted for producing the static magnetic field whose main axis is radial. The bobbin coil is deployed in between the magnet pair, and driven by the transient current for inducing the dynamic electromagnetic field within the pipe. It is also applicable for picking up the EMAT signal i.e., the Electromotive Force (EMF) induced in the coil for fast detection of the external corrosion. The array of induction coils is utilized to acquire the EMAT signals at different angles along the pipe circumference in a bid to monitor the localized integrity of the nonmagnetic pipe. The advantage of the proposed transducer lies in the fact that: (1) The magnet pair and bobbin coil are cylinder-shaped, and able to fit the pipe inner surface, leading to mitigation of the lift-off interference and enhancement of the transduction efficiency; and (2) the generated electromagnetic field including the static and dynamic magnetic fields, eddy currents and the resulting Lorentz force distributes uniformly over the pipe circumference. This results in the uniform excitation of the incident ultrasonic bulk wave along the pipe circumference, which could improve the inspection efficiency.

In this paper, the feasibility of the proposed transducer for efficient evaluation of external corrosion in small-diameter nonmagnetic pipes is intensively investigated via simulations and experiments. The rest of the paper is organized as follows: Section 2 elaborates a 3D hybrid model for efficient computation of testing signals from the proposed transducer. The subsequent simulations for verification of the established hybrid model and investigation regarding the EMAT responses to external corrosion in nonmagnetic pipes are presented in Section 3. This is followed by experimental investigation presented in Section 4.

## 2. 3D Hybrid Model of the Proposed Transducer

The physical phenomena underlying the inspection of nonmagnetic structures using EMATs are constituted by electromagnetics and structural mechanics. It is noteworthy that the excitation of ultrasonic field in the pipe and the resulting EMAT signal are based on electromagnetics whilst the interaction between the generated ultrasonic field and external corrosion is analyzed via structural mechanics. Even though Finite Element Modeling (FEM) concerning both electromagnetics and structural mechanics is prevalent for EMAT simulations [8,10], it is prone to: (1) Considerably high computation burden, leading to time-consuming simulations especially for solutions to the electromagnetic field (extremely dense mesh applied for discretizing the conductor region); and (2) less computation accuracy due to a number of model simplifications regarding the transducer components involving permanent magnets and excitation coils. In order to mitigate the issue, researchers have proposed various models especially the hybrid models for fast simulations of EMATs. Xie et al. established a wholly analytical model of an EMAT array [13] and hybrid models integrating FEM and the Dodd and Deeds’ model for solutions to the electromagnetic field with Finite-difference Time-domain (FDTD) simulations of the UT field [14,15]. This opens up the establishment of a 3D hybrid model of the proposed transducer for the efficient prediction of EMAT signals and transducer optimization.

**Figure 2:**
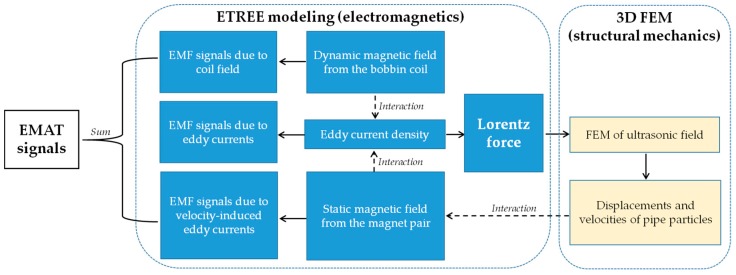
Schematic illustration of the 3D hybrid model.

In the presence of external corrosion symmetrical characteristics of the ultrasonic field can barely hold, the 3D model in lieu of 2D model particularly regarding the ultrasonic simulation is preferred. However, because the excitation frequency of EMATs is normally over 1 MHz, the electromagnetic field and excitation source of the ultrasonic field (i.e., the Lorentz force) in the nonmagnetic pipe are concentrated over the pipe inner surface, and hardly perturbed by the external corrosion. Therefore, the analytical modeling is applicable for fast and accurate solutions to the related electromagnetic quantities. In light of this, a 3D hybrid model integrating the analytical modeling i.e., the Extended Truncated Region Eigenfunction Expansion (ETREE) modeling for solving electromagnetic problems [16,17,18] with 3D FEM for simulations of the ultrasonic field [8] is proposed. The schematic illustration of the 3D hybrid model is portrayed in Figure 2. It is noted that the advantage of the ETREE modeling over Dodd and Deeds’ model lies in the fact that it replaces the integral term of magnetic vector potential with a series of proper eigenfunctions, saving much computing effort for solutions to electromagnetics without sacrificing accuracy [16].

It is noted that the 3D FEM simulation of the ultrasonic field is governed by the dynamic equilibrium equation (concerning merely the pipe) which is written as $\left[M]\{\ddot{u}\}+[C]\{\dot{u}\}+[K]\{u\}=\{F\right\}$ [8,11], where, [*M*], [*C*] and [*K*] are the matrices of the coordination mass, damping and stiffness of every discretized element in the pipe region, respectively. The load matrix {*F*} computed from ETREE comprises the Lorentz force which results from the interaction between the magnetic field and eddy currents in the pipe, and is imposed on each element node analogous to the pipe particle. The unknown matrix {*u*} solved via FEM gives the resulting displacements of all element nodes/pipe particles. It is subsequently exploited for derivation of the particle velocity matrix {*v*} which involves the velocity of each particle/node $\vec{v}$, $\vec{v}=d\vec{u}/dt$, and is directly related to the prediction of testing signals i.e., EMF induced in the induction coil.

**Figure 3:**
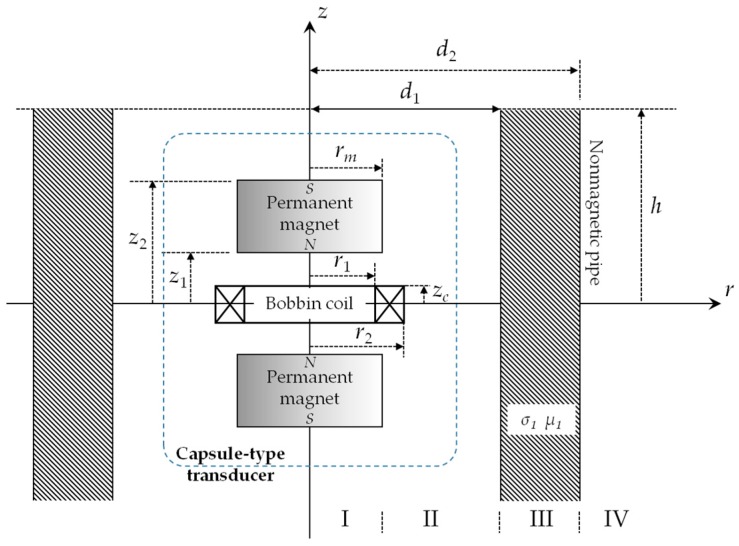
The 2D axi-symmetric model for Extended Truncated Region Eigenfunction Expansion (ETREE) modeling with the proposed capsule-type electromagnetic acoustic transducer.

Compared with full FEM simulations of EMATs, the significant difference of the 3D hybrid model lies in the fact that it employs ETREE modeling for solutions to field quantities related to electromagnetics. Therefore, the 3D hybrid model inherits the advantages of ETREE modeling over FEM in terms of explicit expressions of field quantities, high computational accuracy and speed. This facilitates the theoretical investigation regarding electromagnetic phenomena underlying EMATs and particularly prediction of EMAT signals. The rest of the section is hereby focused on the electromagnetic problems related to the proposed transducer, and elaborates the formulation of closed-form expressions of the field quantities and EMAT signals via ETREE modeling. Since the symmetric characteristics of the electromagnetic field hold regardless of the presence of the external corrosion, the 2D axi-symmetric model for ETREE modeling with the proposed capsule-type EMAT deployed in a nonmagnetic pipe (with the conductivity of *σ*_1_ and relative permeability of $\mu_{1}$, $\mu_{1}=1$) applies, and is presented in Figure 3.

### 2.1. Static Magnetic Field

The static magnetic field is generated from the magnet pair. In conjunction with induced eddy currents in the pipe, it produces the Lorentz force imposed on the pipe particles. The Amperian Current Approach [19] is employed to model the magnet pair analogous to two thin coils with surface currents flowing in opposite directions around the magnet circumference. The surface current density is written as: $J_{m}=\tau_{m}/\left(z_{2}-z_{1}\right)$, where, $\tau_{m}=N_{m}I_{m}$, *I_m_* and *N_m_* are the current intensity and number of turns of the thin coil, respectively. It is noted that *τ_m_* is directly related to the remanence of the magnet *B_rem_* with *τ_m_* = *B_rem_*(*z*_2_ − *z*_1_)/*μ*_0_, where, *μ*_0_ denotes the permeability of vacuum.

Since the *z*-component of the static magnetic field vanishes when *z* = 0, the model of the magnet pair in Figure 3 can be further simplified into the odd parity configuration with only one magnet considered in the solution region. For such case, based on ETREE modeling [16,20], the closed-form expression of the static magnetic field ${\vec{B}}_{sta}$ is formulated as:

(1) $${\vec{B}}_{sta}\left(r,z\right)=\frac{2\mu_{0}r_{m}J_{m}}{h}\sum_{i=1}^{\infty }\left[\cos\left(\alpha_{i}z_{2}\right)-\cos\left(\alpha_{i}z_{1}\right)\right]\left[\begin{array}{l} \cos\left(\alpha_{i}z\right)K_{1}\left(\alpha_{i}r\right){\vec{r}}_{0}\\ +\sin\left(\alpha_{i}z\right)K_{0}\left(\alpha_{i}r\right){\vec{z}}_{0} \end{array}\right]I_{1}\left(\alpha_{i}r_{m}\right)$$

where, ${\vec{r}}_{0}$ and ${\vec{z}}_{0}$ are unit vectors. *α_i_* = *i*π/*h*. *I_n_* and *K_n_* are the modified Bessel functions of first and second kinds respectively. It is noteworthy that the nonmagnetic pipe is transparent within the static magnetic field. Therefore, Equation (1) is applicable for computation of ${\vec{B}}_{sta}$ at an arbitrary position in not only Regions II and IV but also Region III.

### 2.2. Dynamic Electromagnetic Field

The dynamic electromagnetic field involves the dynamic magnetic field and eddy currents induced in the pipe (i.e., Region III in Figure 3). It is generated by the bobbin coil which is concentrically deployed between the magnet pair, and is driven by an excitation current *I*(*t*). Because the *r*-component of the dynamic magnetic field vanishes at *z* = 0, the even parity configuration is utilized for model simplification with half of the bobbin coil taken into account in the solution region. Through ETREE modeling, the closed-form expression of the magnetic vector potential in Region III can be written as:

(2) $$A_{\phi }\left(r,z,t\right)=\frac{\mu_{0}NI\left(t\right)}{d_{1}hz_{c}\left(r_{2}-r_{1}\right)}⊗\sum_{i=1}^{\infty }\left[\cos\left(\beta_{i}z\right)\chi \left(\beta_{i}r_{1},\beta_{i}r_{2}\right)\sin\left(\beta_{i}z_{c}\right)\eta_{1}\left(r,t\right)\right]/\beta_{i}^{3}$$

where, ⊗ denotes the circular convolution. *N* stands for the number of turns of the bobbin coil. *β_i_* = (2*i* − 1)π/(2*h*). The other terms include:

(3) $$\chi \left(x_{1},x_{2}\right)=\int_{x_{2}}^{x_{1}}xI_{1}\left(x\right)dx=\frac{\pi }{2}x\left[L_{0}\left(x\right)I_{1}\left(x\right)-L_{1}\left(x\right)I_{0}\left(x\right)\right]|{}_{x_{2}}^{x_{1}}={\left[2\sum_{k=0}^{\infty }I_{2k+1}(x)-xI_{0}(x)\right]|}_{x_{2}}^{x_{1}}$$

where, *L_n_* stands for the modified Struve function.

(4) $$\left\{ \begin{array}{l} \eta_{1}\left(r,t\right)=\mathrm{FT}\left[\frac{k_{2}K_{1}\left(\lambda_{i}r\right)-k_{4}I_{1}\left(\lambda_{i}r\right)}{k_{1}k_{2}-k_{3}k_{4}}\right]\\ \lambda_{i}=\sqrt{\beta_{i}^{2}+j\omega \mu_{0}\sigma_{1}} \end{array}\right.$$

where, FT denotes the Fourier Transform which can be readily evaluated by using the Fast Fourier Transform (FFT). *ω* is the angular frequency of each harmonic in the excitation current of the bobbin coil. *k*_1_, *k*_2_, *k*_3_ and *k*_4_ are written as:

(5) $$\left\{ \begin{array}{l} k_{1}=\beta_{i}I_{0}\left(\beta_{i}d_{1}\right)K_{1}\left(\lambda_{i}d_{1}\right)+\lambda_{i}I_{1}\left(\beta_{i}d_{1}\right)K_{0}\left(\lambda_{i}d_{1}\right)\\ k_{2}=\beta_{i}I_{1}\left(\lambda_{i}d_{2}\right)K_{0}\left(\beta_{i}d_{2}\right)+\lambda_{i}I_{0}\left(\lambda_{i}d_{2}\right)K_{1}\left(\beta_{i}d_{2}\right)\\ k_{3}=\beta_{i}I_{0}\left(\beta_{i}d_{1}\right)I_{1}\left(\lambda_{i}d_{1}\right)-\lambda_{i}I_{1}\left(\beta_{i}d_{1}\right)I_{0}\left(\lambda_{i}d_{1}\right)\\ k_{4}=\beta_{i}K_{1}\left(\lambda_{i}d_{2}\right)K_{0}\left(\beta_{i}d_{2}\right)-\lambda_{i}K_{0}\left(\lambda_{i}d_{2}\right)K_{1}\left(\beta_{i}d_{2}\right) \end{array}\right.$$

By using the identities $\vec{B}=\nabla \times \vec{A}$ and ${\vec{J}}_{E}=-\sigma \left(\partial \vec{A}/\partial t\right)$ where $\vec{B}$, $\vec{A}$, ${\vec{J}}_{E}$ and *σ* are the magnetic flux density, magnetic vector potential, eddy current density and material conductivity, respectively, the dynamic magnetic field ${\vec{B}}_{dyn}$ and eddy current density *J_e_* at an arbitrary location in Regions III can be formulated as:

(6) $${\vec{B}}_{dyn}\left(r,z,t\right)=\frac{\mu_{0}NI\left(t\right)}{d_{1}hz_{c}\left(r_{2}-r_{1}\right)}⊗\sum_{i=1}^{\infty }\left[\begin{array}{l} \beta_{i}\sin\left(\beta_{i}z\right)\eta_{1}\left(r,t\right){\vec{r}}_{0}\\ +\cos\left(\beta_{i}z\right)\eta_{2}\left(r,t\right){\vec{z}}_{0} \end{array}\right]\frac{\sin\left(\beta_{i}z_{c}\right)\chi \left(\beta_{i}r_{1},\beta_{i}r_{2}\right)}{\beta_{i}^{3}}$$

(7) $$J_{e}\left(r,z,t\right)=\frac{-\sigma_{1}\mu_{0}N}{d_{1}hz_{c}\left(r_{2}-r_{1}\right)}\left\{\frac{\partial \left[I\left(t\right)\right]}{\partial t}\right\}⊗\sum_{i=1}^{\infty }\left[\cos\left(\beta_{i}z\right)\chi \left(\beta_{i}r_{1},\beta_{i}r_{2}\right)\sin\left(\beta_{i}z_{c}\right)\eta_{1}\left(r,t\right)\right]/\beta_{i}^{3}$$

where,

(8) $$\eta_{2}\left(r,t\right)=FT\left[\lambda_{i}\frac{k_{4}I_{0}\left(\lambda_{i}r\right)+k_{2}K_{0}\left(\lambda_{i}r\right)}{k_{3}k_{4}-k_{1}k_{2}}\right]$$

### 2.3. Lorentz Force

The Lorentz force which is imposed on the pipe particles includes two forces i.e., ${\vec{F}}_{s}$ and ${\vec{F}}_{d}$ arising from interactions of induced eddy currents with the static and dynamic magnetic fields, respectively. Based on Equations (1), (6) and (7), the closed-form expression of the Lorentz force on the pipe particle at an arbitrary position in Region III can be written as:

(9) $${\vec{F}}_{s}\left(r,z,t\right)={\vec{J}}_{e}\left(r,z,t\right)\times {\vec{B}}_{sta}\left(r,z\right)=2d_{1}W\left(r,z,t\right)\left\{\begin{array}{l} -\left[\sum_{i=1}^{\infty }\vartheta_{i}\sin\left(\alpha_{i}z\right)K_{0}\left(\alpha_{i}r\right)\right]{\vec{r}}_{0}\\ +\left[\sum_{i=1}^{\infty }\vartheta_{i}\cos\left(\alpha_{i}z\right)K_{1}\left(\alpha_{i}r\right)\right]{\vec{z}}_{0} \end{array}\right\}$$

(10) $${\vec{F}}_{d}\left(r,z,t\right)={\vec{J}}_{e}\left(r,z,t\right)\times {\vec{B}}_{dyn}\left(r,z,t\right)=W\left(r,z,t\right)\left\{\begin{array}{l} -\left[I(t)⊗\sum_{i=1}^{\infty }\zeta_{i}\cos\left(\beta_{i}z\right)\eta_{2}(r,t)\right]{\vec{r}}_{0}\\ +\left[I(t)⊗\sum_{i=1}^{\infty }\zeta_{i}\beta_{i}\sin\left(\beta_{i}z\right)\eta_{1}(r,t)\right]{\vec{z}}_{0} \end{array}\right\}$$

where,

(11) $$\left\{ \begin{array}{l} \vartheta_{i}=r_{m}J_{m}\left[\cos\left(\alpha_{i}z_{2}\right)-\cos\left(\alpha_{i}z_{1}\right)\right]I_{1}\left(\alpha_{i}r_{m}\right)\\ W\left(r,z,t\right)=\sigma_{1}{\left[\mu_{0}^{}/\left(d_{1}h\right)\right]}^{2}\frac{\partial \left[I\left(t\right)\right]}{\partial t}⊗\sum_{i=1}^{\infty }P_{i}\\ P_{i}=\zeta_{i}\cos(\beta_{i}z)\eta_{1}(r,t)\\ \zeta_{i}=\frac{N\sin\left(\beta_{i}z_{c}\right)\chi \left(\beta_{i}r_{1},\beta_{i}r_{2}\right)}{z_{c}\left(r_{2}-r_{1}\right)\beta_{i}^{3}} \end{array}\right.$$

### 2.4. EMF Signals

**Figure 4:**
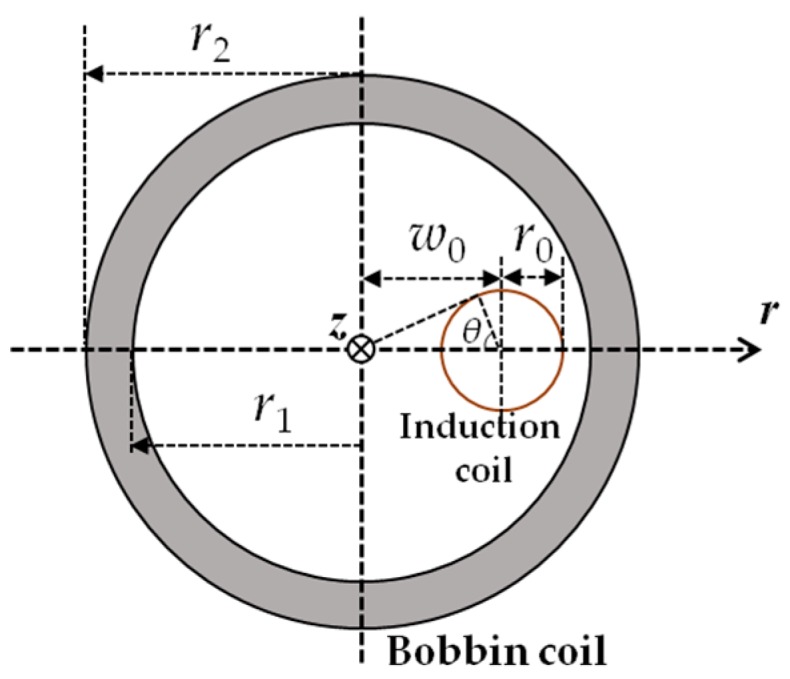
Top view of the bobbin coil and induction coil.

The EMAT signal originates essentially from 3 sources: (1) EMF signals resulting from the dynamic magnetic field from the bobbin coil, *U_d_*; (2) EMF signals due to induced eddy currents in the pipe, *U_e_*; and (3) EMF signals due to velocity-induced eddy currents in the pipe, *U_v_*. As a result, it can readily be predicted by superimposing *U_d_*, *U_e_* and *U_v_*. Note that compared with *U_v_* taking place during the entire inspection including the “exciting” and “listening” stages of the transducer, *U_d_* and *U_e_* occur mostly at the “exciting” stage when the bobbin coil is supplied with the tone-burst current, and vanish at the transducer “listening” stage. As exhibited in Figure 4, for an induction coil (the thin coil with the outer radius of *r*_0_, height of *z_in_*, *z_in_* = 2*z_c_* and number of turns of *N_in_*) deployed at *r* = *w*_0_ within the bobbin coil, based on ETREE modeling the closed-form expressions of *U_d_* and *U_e_* can be formulated as:

(12) $$U_{d}\left(t\right)=4r_{0}\mu_{0}N_{in}\frac{\partial I(t)}{\partial t}\sum_{i=1}^{\infty }\frac{\left(1-2\kappa_{i}z_{c}-2e^{-2\kappa_{i}z_{c}}-1\right)\delta_{i}}{{\left[hJ_{0}(\kappa_{i}h)\right]}^{2}\kappa_{i}^{7}}\int_{0}^{\pi }J_{1}\left[\kappa_{i}\sqrt{r_{0}{}^{2}+w_{0}{}^{2}-2w_{0}r_{0}\cos\theta }\right]d\theta$$

(13) $$U_{e}\left(t\right)=-2r_{0}\mu_{0}N_{in}\frac{\partial I(t)}{\partial t}⊗\sum_{i=1}^{\infty }\frac{\sin(\beta_{i}z_{c})\eta_{1}(t)\zeta_{i}{}^{}}{h\beta_{i}{}^{3}}\int_{0}^{\pi }I_{1}\left[\beta_{i}\sqrt{r_{0}{}^{2}+w_{0}{}^{2}-2w_{0}r_{0}\cos\theta }\right]d\theta$$

In Equation (12), *J_n_* denote the Bessel function. *κ_i_* is the positive root of *J*_1_(*κ_i_h*) = 0. The other term includes:

(14) $$\delta_{i}=\frac{N\varepsilon (\kappa_{i}r_{1},\kappa_{i}r_{2})}{2z_{c}(r_{2}-r_{1})}$$

where, $\varepsilon (x_{1},x_{2})=\int_{x_{1}}^{x_{2}}xJ_{1}(x)dx$ which can be computed referring to [20,21]. The integrals in Equations (12) and (13) can be numerically evaluated using the algorithm presented in [22,23].

During the inspection of the nonmagnetic pipe using the proposed transducer, the ultrasonic wave is generated and propagates through the pipe body. It gets reflected when encountering the material discontinuities involving: (1) The external surface of the pipe; and (2) the bottom of the external corrosion. The generated and reflected ultrasonic waves vibrate each pipe particle with the velocity of $\vec{v}$. In the presence of the static magnetic field, the so-called velocity-induced eddy current is induced, which is constituted by the identity:

(15) $$J_{v}=\sigma_{1}\left(\vec{v}\times {\vec{B}}_{sta}\right)$$

where *J_v_* denotes the density of the velocity-induced eddy currents at the particle position. $\vec{v}$ is derived from FEM. The velocity-induced eddy current subsequently gives rise to *U_v_*. In the presence of the external corrosion, the reflected ultrasonic wave and resulting velocity-induced eddy current are barely uniform over the pipe circumference. In light of this, based on the continuity principle of electric current, special treatment by averaging the calculated *J_v_* at the particle position over the pipe circumference is employed. In such case, a filament comparing a series of pipe particles circumferentially distributing in the pipe applies with the velocity-induced eddy current density equal to the averaged *J_v_*, i.e., $J_{v}^{\prime }$. Based on ETREE modeling, the EMF signal arising from the vibration of a filament in the pipe with the coordinates of *r*_0_ and *z*_0_ is thus written as:

(16) $$U_{v}^{\prime }\left(t\right)=-\frac{4\mu_{0}r_{0}d_{0}N_{in}}{h}\frac{\partial J_{v}^{\prime }(d_{0},z_{0})}{\partial t}\sum_{i=1}^{\infty }\frac{\sin(\beta_{i}z_{c})\cos(\beta_{i}z_{0})K_{1}(\beta_{i}d_{0})}{\beta_{i}}\int_{0}^{\pi }I_{1}\left(\beta_{i}\sqrt{r_{0}{}^{2}+w_{0}{}^{2}-2w_{0}r_{0}\cos\theta }\right)d\theta$$

Following Equation (16), *U_v_* can be readily formulated by superposition of $U_{v}^{\prime }\left(t\right)$ by considering all particles over the pipe inner surface as:

(17) $$U_{v}\left(t\right)=-\frac{4\mu_{0}r_{0}d_{0}N_{in}}{h}\sum_{i=1}^{\infty }\frac{\sin(\beta_{i}z_{c})K_{1}(\beta_{i}d_{0})}{\beta_{i}}\int_{0}^{h}\cos(\beta_{i}z_{0})\frac{\partial J_{v}^{\prime }(d_{0},z_{0})}{\partial t}dz_{0}\int_{0}^{\pi }I_{1}\left(\beta_{i}\sqrt{r_{0}{}^{2}+w_{0}{}^{2}-2w_{0}r_{0}\cos\theta }\right)d\theta$$

By using Equations (12), (13) and (17), the testing signal from the induction coil *ψ*(*t*) can be readily computed via *ψ*(*t*) = *U_d_*(*t*) + *U_e_*(*t*) + *U_v_*(*t*).

In addition to the induction coil, the bobbin coil can also be adopted for acquisition of the EMAT signal which involves EMF induced within the bobbin coil. For such case, Equations (12), (13) and (17) are still applicable whilst the induction coil is presumed to overlap the bobbin coil. Therefore, Equations (12), (13) and (17) can be simplified and rewritten as:

(18) $$U_{d}^{bobbin}\left(t\right)=4\pi \mu_{0}N^{2}\frac{\partial I(t)}{\partial t}\sum_{i=1}^{\infty }\frac{\left(1-2\kappa_{i}z_{c}-2e^{-2\kappa_{i}z_{c}}\right)\delta_{i}\varepsilon (\kappa_{i}r_{1},\kappa_{i}r_{2})}{{\left[hJ_{0}(\kappa_{i}h)\right]}^{2}\kappa_{i}^{7}}$$

(19) $$U_{e}^{bobbin}\left(t\right)=-2\pi \mu_{0}N\frac{\partial I(t)}{\partial t}⊗\sum_{i=1}^{\infty }\frac{\sin(\beta_{i}z_{c})\eta_{1}(t)\chi (\beta_{i}r_{1},\beta_{i}r_{2})\zeta_{i}}{h\beta_{i}{}^{3}}$$

(20) $$U_{v}^{bobbin}\left(t\right)=-\frac{4\pi \mu_{0}d_{0}N}{h}\sum_{i=1}^{\infty }\frac{\sin(\beta_{i}z_{c})K_{1}(\beta_{i}d_{0})\chi (k_{i}r_{1},k_{i}r_{2})}{\beta_{i}^{3}}\int_{0}^{h}\cos(\beta_{i}z_{0})\frac{\partial J_{v}^{\prime }(d_{0},z_{0})}{\partial t}dz_{0}$$

The testing signal from the bobbin coil can thus be predicted by taking the sum of the computed results of $U_{d}^{bobbin}\left(t\right)$, $U_{e}^{bobbin}\left(t\right)$ and $U_{v}^{bobbin}\left(t\right)$ via Equations (18)–(20).

## 3. Corroboration and Simulations

### 3.1. Verification of the Hybrid Model

**Figure 5:**
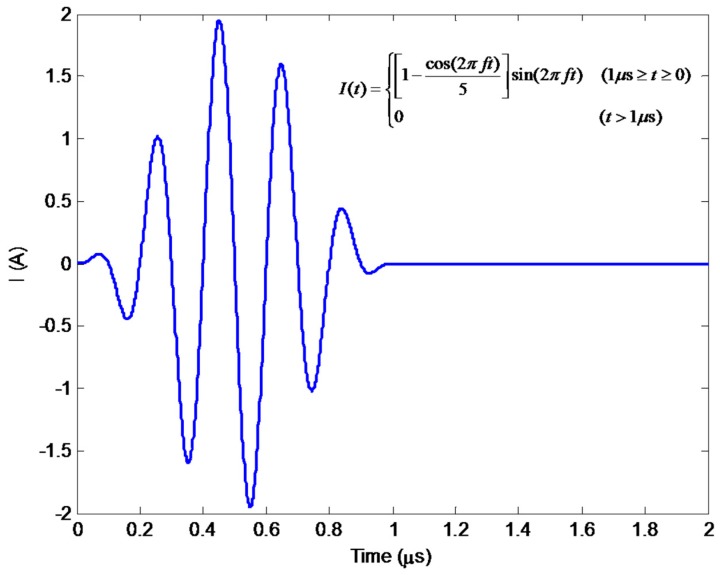
The excitation current with the excitation frequency of 5 MHz for driving the proposed transducer.

**Figure 6:**
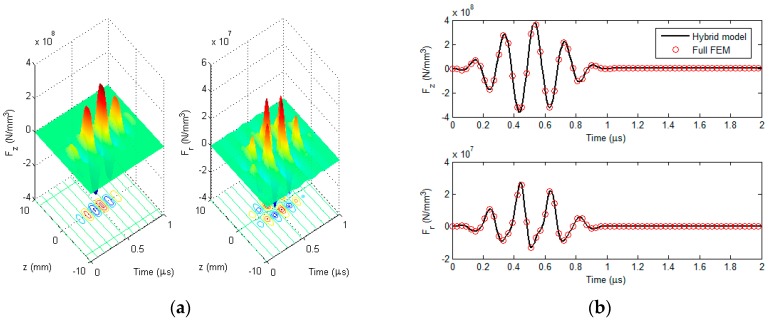
The computed Lorentz force: (**a**) The computed *z*-component and *r*-component of the Lorentz force in the pipe (*r* = 16.01 mm) vs. the axial distance (−10 mm ≤ *z* ≤ 10 mm); and (**b**) the Lorentz force (at *r* = 16.01 mm, *z* = 0.5 mm) computed via the hybrid model and full FEM.

**Figure 7:**
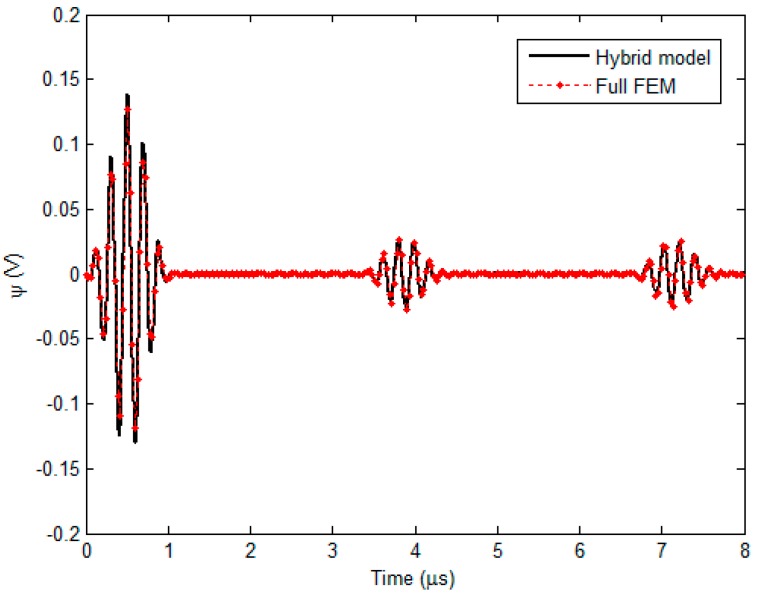
Comparison of the predicted EMAT signal between the hybrid model and full FEM.

Before the hybrid model is exploited for simulations regarding detection and evaluation of external corrosion with the proposed transducer, it is corroborated by using the full FEM simulation [11,24] where the electromagnetic field, ultrasonic field and resulting signal from the induction coil are calculated without the analytical modeling involved. Note that in a bid to obtain simulation results without much loss in accuracy, in full FEM simulations the number of hexahedral mesh elements is up to 1 × 10^7^ whilst the convergence error is set as 1 × 10^−6^. The parameters of the transduer and nonmagnetic pipe are listed in Table 1 and Table 2. The permanent magnet is the N38-type NdFeB magnet. The excitation current (with the excitation frequency of 5 MHz and tone burst width of 1 μs) driving the bobbin coil is presented in Figure 5. The computed *r*-component and *z*-component of the Lorentz force (i.e., *F_r_* and *F_z_*) in the pipe (*r* = 16.01 mm) against the axial distance *z* (−10 mm ≤ *z* ≤ 10 mm) are shown in Figure 6a. It is noteworthy that each component of the Lorentz force is the sum of the corresponding components of ${\vec{F}}_{s}$ and ${\vec{F}}_{d}$. The Lorentz forces at a position (*r* = 16.01 mm, *z* = 0.5 mm) in the pipe which are predicted using the hybrid model and full FEM are compared and exhibited in Figure 6b. The comparison of the resulting signal from the induction coil via the hybrid model with that via full FEM is shown in Figure 7.

It can be observed from Figure 6 that *F_z_* is approximately 10 times higher than *F_r_*, which indicates that the transverse wave dominates in the generated ultrasonic field, and thus the proposed transducer can be regarded as a transverse-wave transducer. It can also be seen from Figure 6b and Figure 7 that the computed Lorentz force and EMAT signal from the hybrid model have good agreement with those from the full FEM. Further analysis reveals that the maximum relative error is up to 2.1%. This implies that the hybrid model is capable of predicting the EMAT signal without much loss in accuracy. Regarding the computation time, it takes 10.8 h for the full FEM to predict the EMAT signal, since the extremely dense mesh should be used for numerical computation of electromagnetic field particularly in the pipe region. In contrast, the computation via the hybrid model costs merely 492 s. The efficient prediction of EMAT signals by using the hybrid model is attributed to the closed-form expressions of related electromagnetic quantities, which are formulated based on ETREE modeling. The established 3D hybrid model is thus beneficial to the efficient simulations with the proposed transducer and transducer optimization.

### 3.2. Simulations with External Corrosion

**Figure 8:**
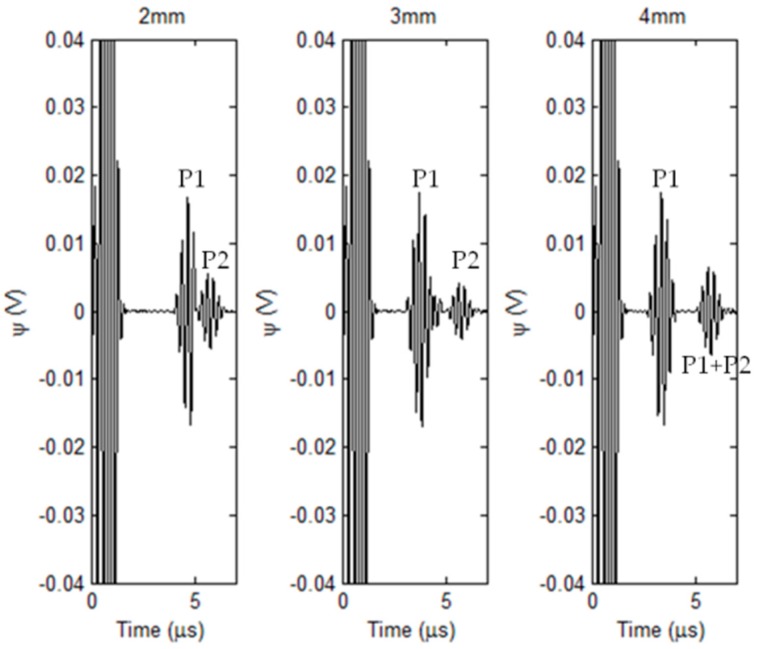
Simulated EMAT signals vs. corrosion depth.

The localized corrosion occurring in the external surface of the nonmagnetic pipe is taken into account in simulations based on the hybrid model. Since among the corrosion parameters, the corrosion depth is the most pressing concern regarding the pipe failure, therefore in simulations the corrosion profile is set as a cylinder with the outer diameter fixed at 12 mm and depth varying from 1 mm to 4 mm. The nominal thickness of the pipe is 8 mm. The other parameters are same as those listed in Table 1 and Table 2. The EMAT signal is computed when the induction coil is right over the external corrosion. The predicted EMAT signal against the corrosion depth is shown in Figure 8.

It can be seen from Figure 8 that the ultrasonic waves reflected from the material discontinuities introduced by the corrosion and external surface of the pipe are indicated by pulses/echos in EMAT signals: (1) P1 for the pulse due to the presence of corrosion; and (2) P2 for the pulse due to the pipe external surface. Similar to traditional UT, the temporal span between P1 and P2 varies with the corrosion depth, and increases when the corrosion depth rises. This implies that: (1) the mechanism of the pipe inspection by using the proposed transducer complies with the basic principal of *UT* for the wall-thickness measurement; and (2) the temporal span between P1 and P2 could be taken as the signal feature for assessment of the corrosion depth. It is noteworthy from Figure 8 that for the corrosion with the depth of 4 mm the pulse after P1 is marked as “P1 + P2”. This is because the corrosion depth is half of the pipe wall thickness, and thus the pulse “P1 + P2” results from the superposition of pulses due to the wave reflections from the corrosion and pipe outer surface.

**Figure 9:**
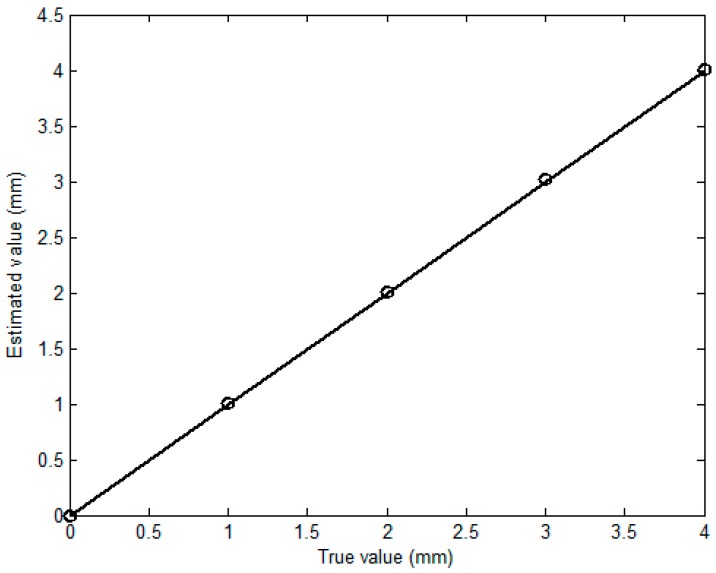
Comparison between the approximated corrosion depths and true values.

In regard to evaluation of the corrosion depth, the arrival times of P1 and P2 are firstly determined by finding the time instant when the magnitude of the envelope of each pulse reaches maximum. The temporal span is subsequently derived from subtraction of the arrival times regarding P1 and P2. The depth of each detected corrosion is thus approximated by computing the product of the temporal span and propagation velocity of transverse wave in the pipe material (3130.8 m/s). The comparison of the corrosion depth between the approximated and true/predefined values is exhibited in Figure 9. It is noticeable from Figure 9 that the approximated depth of each corrosion has good agreement with the true value. The relative error is less than 1.7%. This indicates that the proposed transducer is applicable for detection and depth evaluation of the external corrosion. The simulation results are also supportive of the validity of the proposed hybrid model, since the temporal span extracted from the predicted signal offers good assessment in regard to the corrosion depth without much loss in accuracy.

## 4. Experiments

**Figure 10:**
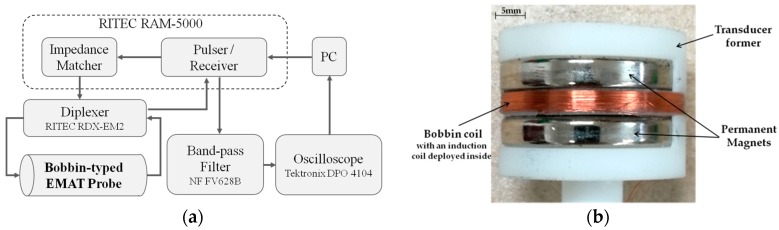
Experimental setup: (**a**) Schematic illustration; and (**b**) picture of the fabricated transducer.

In a bid to further investigate the feasibility of the proposed transducer in detection and evaluation of external corrosion in nonmagnetic pipes, a series of experiments are carried out. The schematic illustration of the experimental system is portrayed in Figure 10a. It is built up based on the commercial EMAT system (RITEC Advanced Measurement System RAM5000, RITEC, INC. 60 Alhambra Road Suite 5, Warwick, RI, US) which is connected with the fabricated transducer presented in Figure 10b. The parameters of the transducer are same as those listed in Table 1. It is noted that for feasibility investigation of the proposed transducer in evaluation of the pipe external corrosion, only one induction coil is deployed in the transducer and used for acquisition of the testing signals. The maximum amplitude of the input voltage (with the same waveform, tone burst width and excitation frequency as those of the excitation current used in simulations) driving the bobbin coil is 50 V. The sampling frequency and gain of the system is 500 MHz and 70 dB, respectively. The Signal-to-Noise Ratio (SNR) of the measurement can reach approximately 35 dB by averaging the acquired EMAT signal for 512 times.

**Figure 11:**
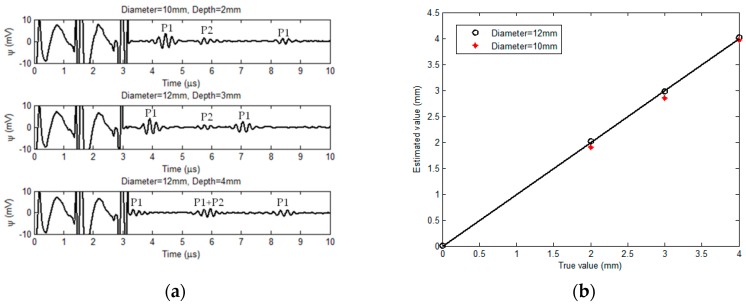
Experimental results: (**a**) Acquired signals vs. corrosion sizes; and (**b**) comparison between the approximated corrosion depths and true values.

The sample adopted in the experiments is an aluminum-alloy pipe with the inner radius and nominal thickness of 16 mm and 8 mm, respectively. Flat-bottom holes are fabricated on the outer surface of the sample to simulate the external corrosion. The diameters of the flat-bottom hole are 10 mm and 12 mm whilst the hole depth varies from 2 mm to 4 mm. It is noted that the diameter of 12 mm along with the depth of 4 mm is critical size of the external corrosion for maintenance/replacement of the pipe under inspection. During experiments, the proposed transducer scans over the pipe inner surface. The acquired EMAT signals when the induction coil is right over the external corrosion are shown in Figure 11a.

As can be seen from Figure 11a, by using the proposed transducer the external corrosion particularly the one in the critical size can be detected, the presence of which is indicated by P1 in the EMAT signal. The temporal span between P1 and P2 in the acquired signal from the proposed transducer is highly dependent of the corrosion depth whilst it is barely sensitive to the corrosion diameter. Further analysis reveals that the temporal span is directly proportional to the corrosion depth. This is supportive of the findings from the simulations. By applying the same signal processing method in simulations, the temporal span corresponding to each corrosion is extracted from the experimental signal, and subsequently adopted for estimation of the corrosion depth in conjunction with the velocity of transverse-wave propagation in the pipe material. The comparison between estimated corrosion depths and true values is presented in Figure 11b. Good agreement of the estimated corrosion depth with the true value can be found from Figure 11b with the relative error less than 4.9%. Although the extraneous noise in experiments and small eccentricity of the transducer in the sample make the maximum relative error from the experiment slightly higher than that from the simulation, the experimental results further reveal the feasibility of the proposed transducer in quantitative evaluation of external corrosion in nonmagnetic pipes.

**Figure 12:**
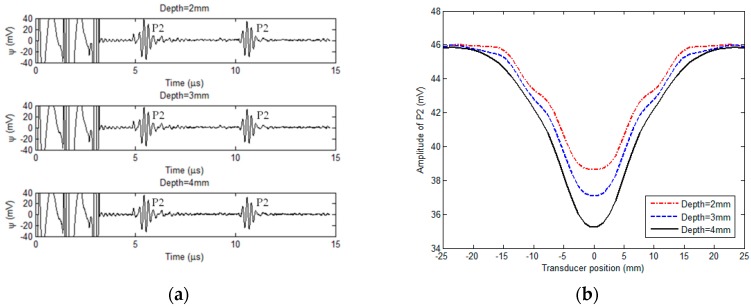
Experimental results with the bobbin coil for signal acquisition: (**a**) Acquired signals vs. corrosion depths; and (**b**) scanning curves for corrosion in different depths.

Special attention has been given to the amplitudes of P1 and P2 in the EMAT signal. It is noticeable from Figure 11a that for each corrosion scenario the amplitude of P1 is higher than that of P2. At the position of the induction coil, stronger reflection of the ultrasonic wave occurs at the corrosion bottom than that at the pipe external surface. As a result, the ultrasonic wave reflected from the corrosion gives the dominant contribution to the EMAT signal compared with the wave from the pipe external surface, leading to higher amplitude of P1 than that of P2. The analysis indicates that in the presence of corrosion, the magnitude of P2 decreases due to wave reflection from the corrosion. Since the bobbin coil in the proposed transducer can also be used for acquisition of testing signals, the external corrosion could be detected by analyzing the P2 amplitude of the signal from the bobbin coil, which is complementary to the corrosion detection by using the signal from the induction coil. For the corrosion with the depth varying from 2 mm to 4 mm and diameter fixed at 10 mm, the acquired signals from the bobbin coil are exhibited in Figure 12a.

From Figure 12a, it can be seen that P2 resulting from the wave reflection from the pipe external surface can be observed. The two identified pulses marked as P2 in each testing signal indicate the ultrasonic wave bouncing back and forth within the pipe. In contrast, P1 depicting the ultrasonic wave reflected from the corrosion can hardly be identified. This is because: (1) The bobbin coil essentially monitors the averaged wall thickness around the pipe circumference and the contribution from the corrosion to the wall thinning is feeble; and (2) the extraneous noise as well as strong amplitude of P2 can severely mask P1. However, since the presence of the corrosion leads to the suppression regarding the wave reflection from the pipe external surface, the corrosion can be indirectly detected by analyzing the magnitude of P2. The scanning curve of the P2 magnitude against the transducer position for each corrosion is shown in Figure 12b. It can be observed from Figure 12b that the amplitude of P2 in the signal from the bobbin coil decreases when the proposed transducer is positioned at the corrosion region. It hits the trough when the bobbin coil in the transducer is right over the corrosion center. The minimum value of the scanning curve is inversely proportional to the corrosion depth which could be further evaluated by using the calibration method. The experimental results indicate that the bobbin coil of the proposed transducer could also be applicable for detection of the external corrosion. Compared with the induction coil, the application of the bobbin coil for the signal acquisition may facilitate the fast screening of external corrosion. During the inspection of nonmagnetic pipes, the proposed transducer scans over the pipe inner surface in both axial and circumferential directions. The signal from the bobbin coil could be adopted for fast detection of the external corrosion and determination of its axial location. The signal from the induction coil is subsequently used for: (1) Further identification of the circumferential and axial positions of the corrosion; and (2) dedicated evaluation of the detected corrosion particularly regarding the corrosion depth.

## 5. Concluding Remarks

In this paper, a capsule-type EMAT is proposed for detection and evaluation of external corrosion in nonmagnetic pipes. It consists of a concentric magnet pair, a bobbin coil and induction coils, and facilitates the excitation of the incident ultrasonic field uniformly distributing around the pipe circumference. The feasibility of the proposed transducer in evaluation of external corrosion is investigated via simulations and experiments.

A 3D hybrid model integrating the ETREE modeling for efficient computation of electromagnetic quantities with FEM intensively simulating the ultrasonic field is established in regard to the proposed transducer. The closed-form expressions of the electromagnetic quantities and testing signals from the bobbin coil and induction coil are formulated. The verification of the hybrid model with full FEM indicates the hybrid model is valid and capable of efficiently predicting testing signals from the proposed transducer. Following this, simulations based on the hybrid model are conducted. It has been found from the simulation results that the temporal span between P1 and P2 within the EMAT signal give good implication regarding the presence as well as the depth of the external corrosion. This is supported by the experimental investigation with the fabricated transducer. The experimental signals from the proposed transducer against the external corrosion with various sizes are obtained and further analyzed. It has been found from the experimental results that the testing signal from the induction coil can be adopted for detection of the external corrosion and evaluation of its depth. Good agreement between the estimated and true values regarding the corrosion depth can be identified. In addition, the testing signal from the bobbin coil is also investigated. It is noticeable from the results that the bobbin coil in the proposed transducer could be employed for fast detection of the external corrosion, which would be complementary to the corrosion detection and evaluation by using the induction coil. The investigation through simulations and experiments indicates the feasibility of the proposed capsule-type EMAT in detection and evaluation of external corrosion in nonmagnetic pipes.

Following current work, further research involves: (1) Investigation regarding the influence of corrosion orientation on testing signals from the proposed transducer; and (2) optimization of the capsule-type EMAT and signal processing for detection and quantitative evaluation of natural corrosion and those with small sizes such as the pitting corrosion.

## Figures and Tables

**Table 1:** Parameters of the proposed transducer.

Transducer Parameter	Value
Remanence field of the magnet, *B_rem_* (T)	1.47
Position of the magnet upper surface, *z*_1_ (mm)	2.0
Magnet height, *z*_2_ − *z*_1_ (mm)	5.0
Magnet Radius, *r_m_* (mm)	15.0
Inner radius of the bobbin coil, *r*_1_ (mm)	15.4
Outer radius of the bobbin coil, *r*_2_ (mm)	15.6
Height of the bobbin coil, 2*z_c_* (mm)	3.0
Number of turns of the bobbin coil, *N*	32
Radius of the induction coil, *r*_0_ (mm)	2.0
Height of the induction coil, *z_in_* (mm)	3.0
Number of turns of the induction coil, *N_in_*	30
Distance between the centers of the bobbin coil and induction coil, *w*_0_ (mm)	13.3
Liftoff distance, *d*_1_ − *r*_2_ (mm)	0.4

**Table 2:** Parameters of the nonmagnetic pipe.

Pipe Parameter	Value
Inner radius, *d*_1_ (mm)	16.0
Outer radius, *d*_2_ (mm)	22.0
Conductivity, *σ*_1_ (MS·m^−1^)	34.2
Relative permeability, *μ*_1_	1.0
Young’s modulus (GPa)	71.7
Density (kg·m^−3^)	2750
Poisson’s ratio	0.33
